# Fam20C Overexpression Predicts Poor Outcomes and is a Diagnostic Biomarker in Lower-Grade Glioma

**DOI:** 10.3389/fgene.2021.757014

**Published:** 2021-12-14

**Authors:** Jing Feng, Jinping Zhou, Lin Zhao, Xinpeng Wang, Danyu Ma, Baoqing Xu, Feilai Xie, Xingfeng Qi, Gang Chen, Hu Zhao, Junxin Wu

**Affiliations:** ^1^ Department of Radiation Oncology, The Third Clinical Medical College of Fujian Medical University, Fuzhou, China; ^2^ The 900th Hospital of Joint Logistic Support Force, PLA, Fuzhou, China; ^3^ Department of Clinical Quality Management, The 900th Hospital of Joint Logistic Support Force, PLA, Fuzhou, China; ^4^ Department of Neurosurgery, The 900th Hospital of Joint Logistic Support Force, PLA, Fuzhou, China; ^5^ Department of Radiation Oncology, The 900th Hospital of Joint Logistic Support Force, PLA, Fuzong Clinical Medical College of Fujian Medical University, Fuzhou, China; ^6^ Department of Pathology, The 900th Hospital of Joint Logistic Support Force, PLA, Fuzhou, China; ^7^ Department of Pathology, The 900th Hospital of Joint Logistic Support Force, Fujian Medical University Cancer Hospital, Fujian Cancer Hospital, Fuzhou, China; ^8^ Department of General Surgery, The 900th Hospital of Joint Logistic Support Force, PLA, Fujian Medical University, Fuzhou, China; ^9^ Department of Radiation Oncology, Fujian Medical University Cancer Hospital, The Third Clinical Medical College of Fujian Medical University, Fuzhou, China; ^10^ Fujian Key Laboratory of Translational Cancer Medical, Fujian Cancer Hospital, Fujian Provincial Clinical Research Center for Cancer Radiotherapy and Immunotherapy, Fuzhou, China

**Keywords:** FAM20C, lower-grade gliomas, LGG, biomarker, prognosis, bioinformatics

## Abstract

Glioma is a relatively low aggressive brain tumor. Although the median survival time of patients for lower-grade glioma (LGG) was longer than that of patients for glioblastoma, the overall survival was still short. Therefore, it is urgent to find out more effective molecular prognostic markers. The role of the Fam20 kinase family in different tumors was an emerging research field. However, the biological function of Fam20C and its prognostic value in brain tumors have rarely been reported. This study aimed to evaluate the value of Fam20C as a potential prognostic marker for LGG. A total of 761 LGG samples (our cohort, TCGA and CGGA) were included to investigate the expression and role of Fam20C in LGG. We found that Fam20C was drastically overexpressed in LGG and was positively associated with its clinical progression. Kaplan-Meier analysis and a Cox regression model were employed to evaluate its prognostic value, and Fam20C was found as an independent risk factor in LGG patients. Gene set enrichment analysis also revealed the potential signaling pathways associated with Fam20C gene expression in LGG; these pathways were mainly enriched in extracellular matrix receptor interactions, cell adhesion, cell apoptosis, NOTCH signaling, cell cycle, etc. In summary, our findings provide insights for understanding the potential role of Fam20C and its application as a new prognostic biomarker for LGG.

## Introduction

Malignant central nervous system tumors account for 31.5% of nervous system tumors, and gliomas account for 80.7% of malignant central nervous system tumors (Goodenberger and Jenkins 2012; Ostrom et al., 2018). Global cancer statistics in 2018 showed that nervous system cancer was the 19th most common cancer in the world, with 296,851 new cases, accounting for 1.6% of the total cancer incidence, and 241,037 deaths each year, accounting for 2.5% of the total case mortality (Bray et al., 2018). According to the World Health Organization (WHO) 2016 version of the central nervous system classification, diffuse gliomas include WHO grade II and grade III astrocytic tumors, grade II and III oligodendrogliomas, and grade IV glioblastomas (Louis et al., 2016; Wesseling and Capper 2018).

At present, the standard treatment of glioma includes surgical resection to the maximum safety range followed by postoperative radiotherapy and chemotherapy (Stupp et al., 2005). However, the prognosis of glioma is still poor. Glioblastoma (GBM) is the most aggressive type of brain tumor in adults. Despite the improvement of current treatment methods, the median survival time is only 17–23 months (Xu et al., 2017; Jiang et al., 2019). The median survival time of WHO grade II-III glioblastoma is longer than that of WHO grade IV glioblastoma, with a median survival time of 1.7–13.3 years (Buckner et al., 2016; Mair et al., 2021; van den Bent 2014). There is extensive heterogeneity among lower-grade glioma patients. Some patients could survive for many years without any treatments; however, other patients progress quickly after active treatment. Therefore, it is very important to find more effective molecular prognostic markers for the treatment of patients with LGG. Understanding the pathogenesis and etiology of LGG may assist in discovering advanced treatment methods and effective biomarkers for diagnosis and prognosis.

The Fam20 kinase family is a newly discovered class of secreted kinases that can phosphorylate secreted proteins and proteoglycans. This family includes Fam20A, Fam20B, and Fam20C (Nalbant et al., 2005; Zhang et al., 2018). Fam20C is a casein kinase protein enriched in the Golgi that can phosphorylate a variety of secreted proteins (Tagliabracci et al., 2014; Cozza et al., 2018). Protein phosphorylation modification refers to the process of transferring the phosphate group of ATP or GTP to the amino acid residue of the substrate protein through the catalytic effect of a protein kinase (Fischer 2013). This process mediates most of the signal transduction in eukaryotic cells and it regulates many cellular processes, including metabolic regulation, transcription regulation, cell cycle, cytoskeleton rearrangement, apoptosis, and differentiation (Manning et al., 2002; Sreelatha et al., 2015). Abnormal protein phosphorylation is the leading cause of many diseases, including cancer, diabetes, Alzheimer’s disease, and Parkinson’s disease (Fischer 2013; Klement and Medzihradszky 2017).

Fam20C is located inside the cell, but it may also play an important role outside the cell (Wang et al., 2013; Tagliabracci et al., 2015). Fam20C has been shown to phosphorylate secreted proteins by recognizing the protein motif “Ser-x-Glu/phospho-Ser,” thereby being involved in biomineralization, lipid homeostasis, cell adhesion and migration. More importantly, many Fam20C substrates are related to tumor cell apoptosis and metastasis, including insulin-like growth factor binding proteins, osteopontin, and serine protease inhibitors (Rangaswami et al., 2006; Baxter 2014; Zhang et al., 2020). Insulin-like growth factor binding protein 7 (IGFBP7), which depends on Fam20C phosphorylation, could induce cell migration (Bieche et al., 2004; Georges et al., 2011). However, the utility of Fam20C as a potential tumor diagnostic and prognostic marker has not been fully elucidated.

In this study, we found that Fam20C was overexpressed in a variety of cancers, including LGG. High expression of Fam20C was associated with tumor progression. Therefore, Fam20C may serve as a potential biomarker for the diagnosis and prognosis of LGG. Moreover, the transcriptional expression of Fam20C in LGG patients may be an independent risk factor for survival. In addition, pathway and function enrichment indicated that the mechanism of Fam20C-mediated tumorigenesis involves extracellular matrix receptor interactions, cell adhesion, and the cell cycle. Our results clarified the important role of Fam20C in the prognosis of LGG and provided a reliable biomarker for the diagnosis and prognosis of LGG.

## Materials and Methods

### Data Acquisition and Processing

LGG gene expression data and clinical information were obtained from The Cancer Genome Atlas TCGA database (http://cancergenome.nih.gov/) and the Chinese Glioma Genome Atlas CGGA database (http://www.cgga.org.cn). From the TCGA database, we obtained the original mRNAseq data of 529 LGG samples, which were normalized using the edge R package in R (version 4.0.2). A total of 132 LGG samples were obtained from the CGGA database, and the gene expression profile of each sample and the corresponding clinical data were sorted (Supplementary Table S1). The RNA-seq data from the CGGA database were generated from total RNA and directly expressed as fragment values per thousand bases per million mapped reads (FPKM). In CGGA database, a rapid hematoxylin and eosin-stain for frozen sections was applied to each sample to assess the tumor cell proportion before RNA extraction. In addition, the RNA was extracted from only those samples with >80% tumor cells (Zhao Z. et al., 2021).

### Patient Information and Ethics

This study was approved by the ethics committee of 900th Hospital of Joint Logistics Support Force. Between January 2016 and November 2020, a cohort assessment of 100 patients who underwent neurosurgery was conducted. According to the WHO 2007 and 2016 standards, all patients were newly diagnosed with grade II and III gliomas. Patients younger than 16 years old at the time of diagnosis were excluded from this study (Supplementary Table S1). Clinical data and detailed follow-up data were obtained from all patients. Sanger sequencing was then employed to investigate the mutation status of isocitrate dehydrogenase (IDH). In addition, we also studied the 1p/19q deletion and the heterozygosity status of LGG using fluorescence *in situ* hybridization.

### Immunohistochemistry Analysis

One hundred patients with LGG and three normal brain tissues from grade 1 glioma patients in the 900th Hospital of Joint Logistics Support Force were collected. The adjacent brain tissues to the three cases of grade 1 glioma patients were used as normalized data. The surgical specimens were fixed with 40 g/L formaldehyde solution, routinely embedded in paraffin, cut into 4 μm-thick sections, and stained with HE. The EliVision method was used for Fam20C immunohistochemical staining and the results were observed through light microscopy. Anti-Fam20C polyclonal antibody, was purchased from Abcam, UK (product number ab154740). Non-biotin universal two-step immunohistochemistry kit (mouse/rabbit enhanced polymer detection system) was purchased from Beijing Zhongshan Jinqiao Biotechnology Co., Ltd. The positive control tissue in this experiment was glioblastoma tumor tissues (Du et al., 2020). Results interpretation criteria: Fam20C positive expression means brown-yellow particles in the nucleus and cytoplasm. Dark brown in the nucleus and cytoplasm of the cells was defined as a strong cell; Cells with yellow or brown nucleus and cytoplasm were defined as medium-strength cells; The nucleus and cytoplasm of the cells were light yellow or had faintly visible staining, which was defined as a weak intensity cell. No staining of nucleus and cytoplasm was negative. The histochemical score (H-score) was employed to quantify the expression of Fam20C. H-score = (percentage of weak intensity cells×1) + (percentage of medium intensity cells 2) + (percentage of strong cells×3).

### Gene Set Enrichment Analysis (GSEA)

GSEA was conducted to detect whether a set of a priori defined genes showed statistically significant differential expression between the high and low Fam20C expression groups during the MSigDB set enrichment process, with 1000 genome permutations performed per analysis. In this study, GSEA first generated an ordered list of all genes based on the correlation between the genes and Fam20C expression. Then, GSEA was performed to clarify the significance of the difference in survival between the high and low Fam20C expression groups. The expression level of Fam20C was used as the phenotype label. The phenotypic enrichment pathways were ranked by the nominal *p* value and normalized enrichment score. The calculation results were given using the ggplot2 R packages.

### Functional Enrichment Analysis

Gene Ontology (GO) was employed to detect the function of the differentially expressed genes. The analysis gained a new understanding of the biological effects of Fam20C. The genes related to Fam20C expression (absolute Pearson correlation coefficient>0.5 and *p* < 0.05) were regarded as risk score-related genes, and their potential biological functions and pathways were determined. The Ggplot2 software package in R software was employed to analyze the GO pathways. The enrichment analysis of GO was based on a *p*-value and a q-value threshold <0.05.

### Statistical Analysis

The Wilcoxon signed-rank test was used to detect the expression of Fam20C. The correlation between the clinicopathological characteristics and Fam20C expression was tested with the Wilcoxon signed-rank test. The survival ROC software package in R software was used to generate receiver operating characteristic (ROC) curves to evaluate the diagnostic value of Fam20C expression. The area under the curve represents the diagnostic value. Using the Survival package in R, the overall survival (OS) rates of the high expression group and the low expression group were compared by Kaplan-Meier analysis. Univariate Cox analysis was used to determine the potential survival rate, and multivariate Cox analysis was used to determine whether Fam20C expression was an independent risk factor for OS in LGG patients. *p* < 0.05 was considered statistically significant. All data were processed using R software (version 4.0.2) and Adobe Photoshop CC.

## Results

### Fam20C Was Overexpressed in LGG

**FIGURE 1:**
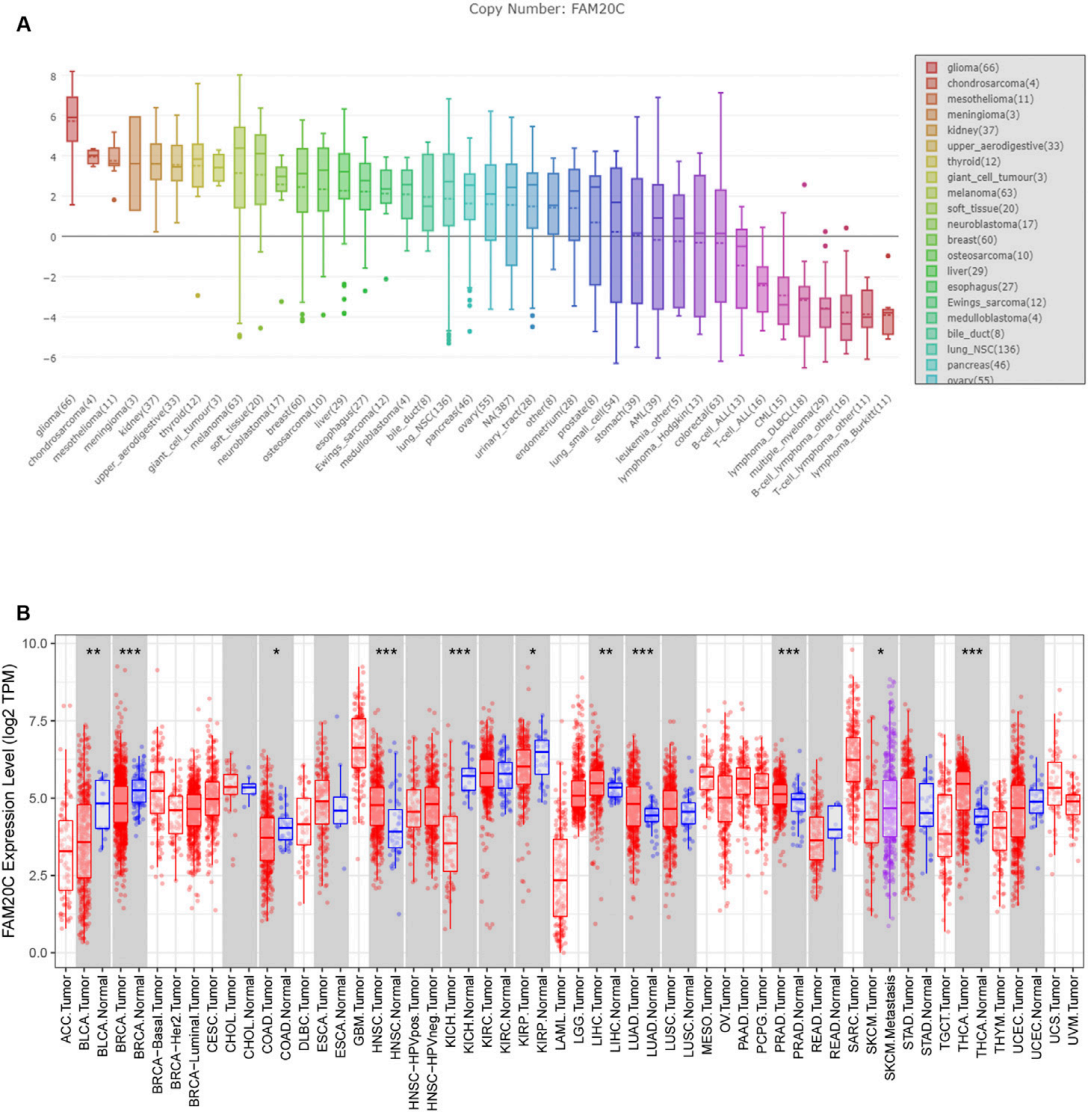
The expression of FAM20C in different types of cancer, including glioma. **(A)** The expression of FAM20C in different types of cancer cells was obtained from the CCLE database, including glioma (*n* = 66), chondrosarcoma (*n* = 4), mesothelioma (*n* = 11), meningioma (*n* = 3), kidney (*n* = 37), upper aerodigestive (*n* = 33), thyroid (*n* = 12), giant cell tumour (*n* = 3), melanoma (*n* = 63), soft tissue (*n* = 20), neuroblastoma (*n* = 17), breast (*n* = 60), osteosarcoma (*n* = 10), liver (*n* = 29), esophagus (*n* = 27), Ewing’s sarcoma (n = 12), medulloblastoma (*n* = 4), bile duct (*n* = 8), lung NSC (*n* = 136), pancreas (*n* = 46), ovary (*n* = 55), urinary tract (*n* = 28), endometrium (*n* = 28), prostate (*n* = 8), lung small cell (*n* = 54), stomach (*n* = 39), acute myeloid leukemia (*n* = 39), leukemia other (*n* = 5), lymphoma Hodgkin (*n* = 13), colorectal (*n* = 63), B cell acute lymphoblastic leukemia (*n* = 13), T cell acute lymphoblastic leukemia (*n* = 16), chronic myelogenous leukemia (*n* = 15), lymphoma DLBCL (*n* = 18), multiple myeloma (*n* = 29), B cell lymphoma other (*n* = 16), T cell lymphoma other (*n* = 11), and lymphoma Burkitt (*n* = 11); **(B)** the expression of FAM20C in different types of cancer was obtained from Tumor Immune Estimation Resource database, including ACC (*n* = 77), BLCA (*n* = 423), BRCA (*n* = 1197), CESC (*n* = 309), CHOL (*n* = 45), COAD (*n* = 316), DLBC (*n* = 47), ESCA (*n* = 195), GBM (*n* = 163), HNSC (*n* = 563), KICH (*n* = 91), KIRC (*n* = 595), KIRP (*n* = 318), LAML (*n* = 173), brain LGG (*n* = 518), LIHC (*n* = 419), LUAD (*n* = 542), LUSC (*n* = 542), MESO (*n* = 87), OV (*n* = 426), PAAD (*n* = 183), PCPG (*n* = 185), PRAD (*n* = 544), READ (*n* = 102), SARC (*n* = 264), SKCM (*n* = 462), STAD (*n* = 444), TGCT (*n* = 137), THCA (*n* = 571), THYM (*n* = 120), UCEC (*n* = 187), UCS (*n* = 57), and UVM (*n* = 79). *p < 0.05; ***p < 0.001.

Data from the Cancer Cell Line Encyclopedia (CCLE) database showed that Fam20C was highly expressed in multiple cancer cell lines, especially glioma (Figure 1A). At present, there are few studies on the relationship between Fam20C and tumorigenesis. To determine the expression of Fam20C in other tumors, we conducted a comprehensive analysis of 33 tumors in TCGA. Among them, there were five cancer types in which Fam20C was overexpressed (Figure 1B).

### Overexpressed Fam20C Was Associated With Advanced LGG

**FIGURE 2:**
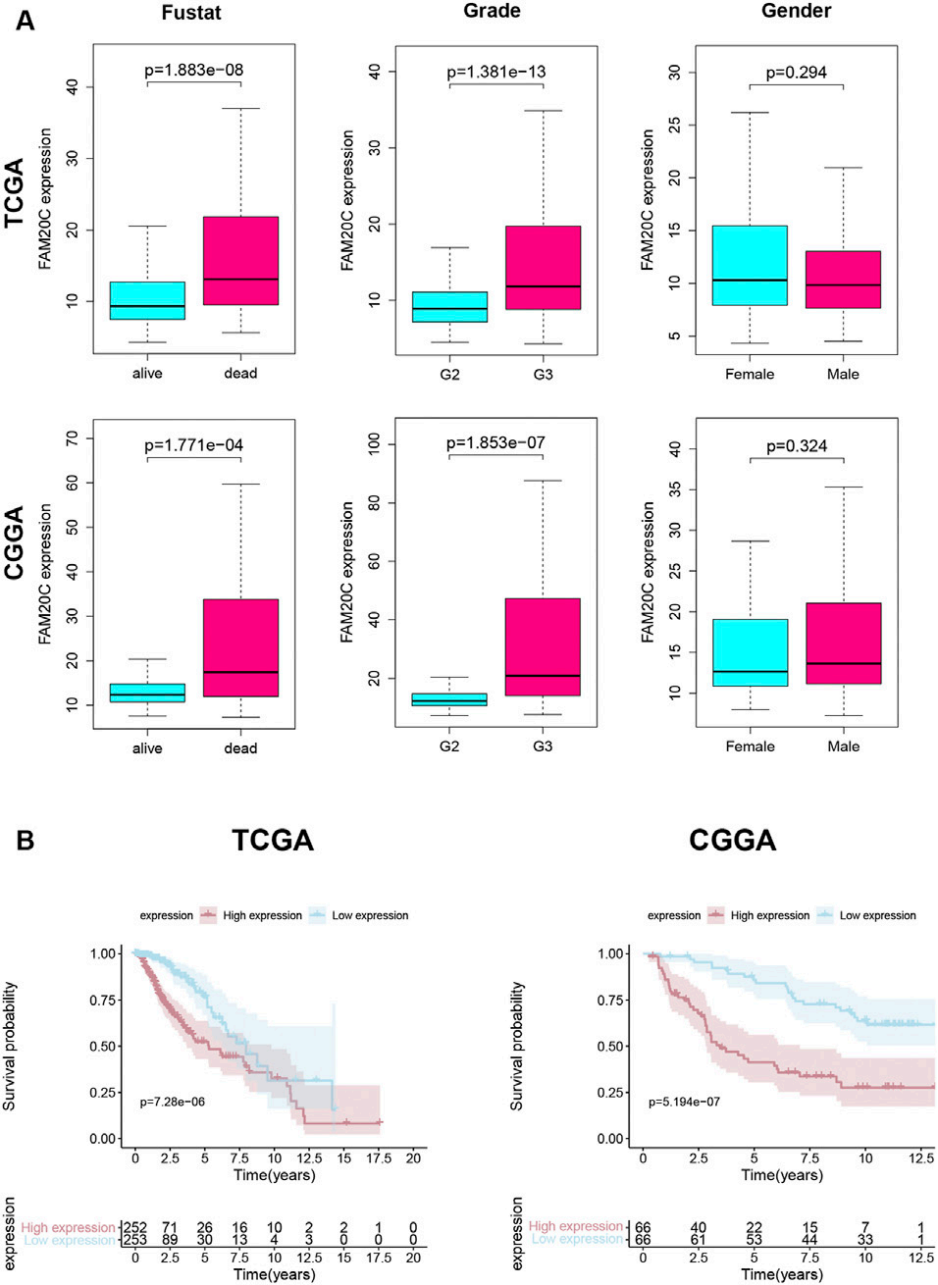
Association with FAM20C expression and clinicopathological characteristics. **(A)** Clinical in TCGA database, including grade (grade 2 *n* = 248, and grade 3 *n* = 261); fustat (alive *n* = 400, and dead *n* = 109); gender (male *n* = 281, and female *n* = 228); Clinical in CGGA database, including grade (grade 2 *n* = 87, and grade 3 *n* = 45); fustat (alive *n* = 68, and dead *n* = 64); gender (male *n* = 81, and female *n* = 51); TCGA, The Cancer Genome Atlas. CGGA, Chinese Glioma Genome Atlas. **(B)** Kaplan–Meier curves for OS in LGG Higher FAM20C expression was remarkably associated with poorer OS in TCGA database; Higher FAM20C expression was remarkably associated with poorer OS in CGGA database. OS, overall survival. The fustat means the patients’ survival status.

Next, we analyzed the correlation between the level of Fam20C mRNA in LGG patients and their clinicopathological parameters. The TCGA database includes the patient’s tumor grade, sex, and survival status. The CGGA database includes the patient’s tumor grade, sex, survival status, IDH mutation/wild-type, and 1p19q joint deletion status. As shown in Figure 2A, the higher the grade of the tumor, the higher the Fam20C expression level. In addition, in the TCGA database, high expression of the Fam20C gene was positively related to grade and survival status but not to sex. In the CGGA database, higher Fam20C expression was related to grade, survival status, IDH wild-type, and 1p19q nonjoint deletion but not to sex (Figure 2A and Supplementary Figure S1).

Since high expression of Fam20C in LGG patients was related to tumor grade, we further tried to determine whether this overexpression of Fam20C in LGG patients was related to a poor prognosis through the use of Kaplan-Meier curves. As shown in Figure 2B, higher Fam20C expression levels were significantly correlated with a worse OS in both the TCGA and CGGA datasets (Figure 2B). In general, the results showed that the expression of Fam20C was significantly related to the prognosis of LGG patients and could be used as a biomarker to predict the survival of LGG patients.

### High Fam20C Expression Served as an Independent Risk Factor Among LGG Patients

**FIGURE 3:**
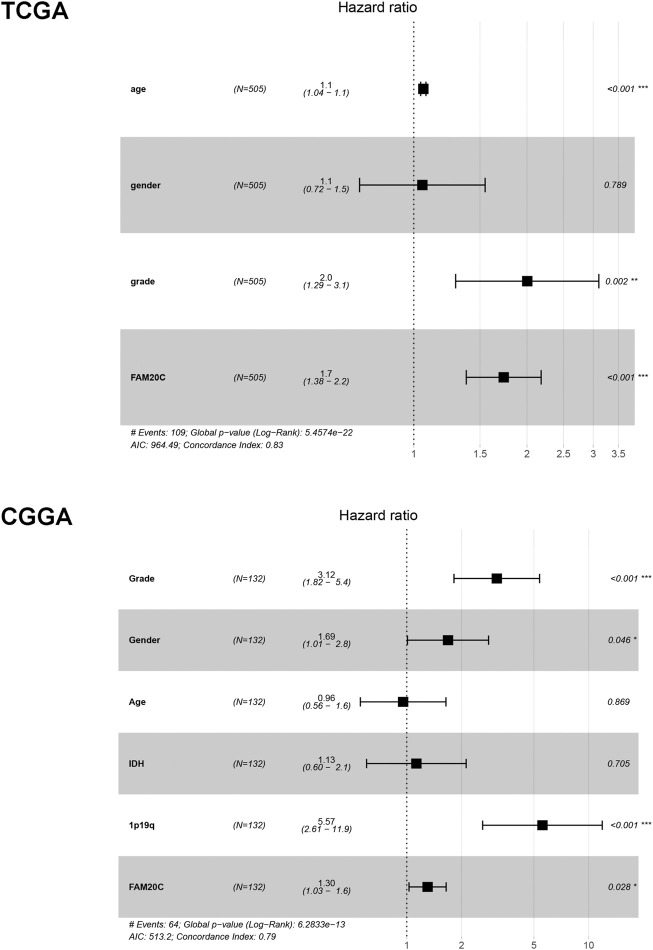
Multivariate Cox analysis evaluating independently predictive ability of Fam20c for OS in TCGA and CGGA database. **p < 0.01; ***p < 0.001.

Univariate and multivariate Cox analyses were utilized to evaluate the independent prognostic values of Fam20C expression in LGG patients. The univariate analysis results showed that high Fam20C expression was significantly correlated with a shorter OS (HR = 1.02, 95% CI: 1.01–1.03, *p* < 0.001; HR = 1.01, 95% CI: 1.00–1.01, *p* = 0.001) in TCGA and CGGA. Other variables related to poor survival included age and grade in TCGA (Supplementary Table S2). In CGGA, variables related to poor survival that including grade IDH and 1p19q (Supplementary Table S3). Multivariate analysis showed that high expression of Fam20C in LGG patients was independently associated with a significant decrease in OS (Figure 3 and Supplementary Tables S2, S3).

### Fam20C Expression Is a Novel Diagnostic Biomarker for LGG

**FIGURE 4:**
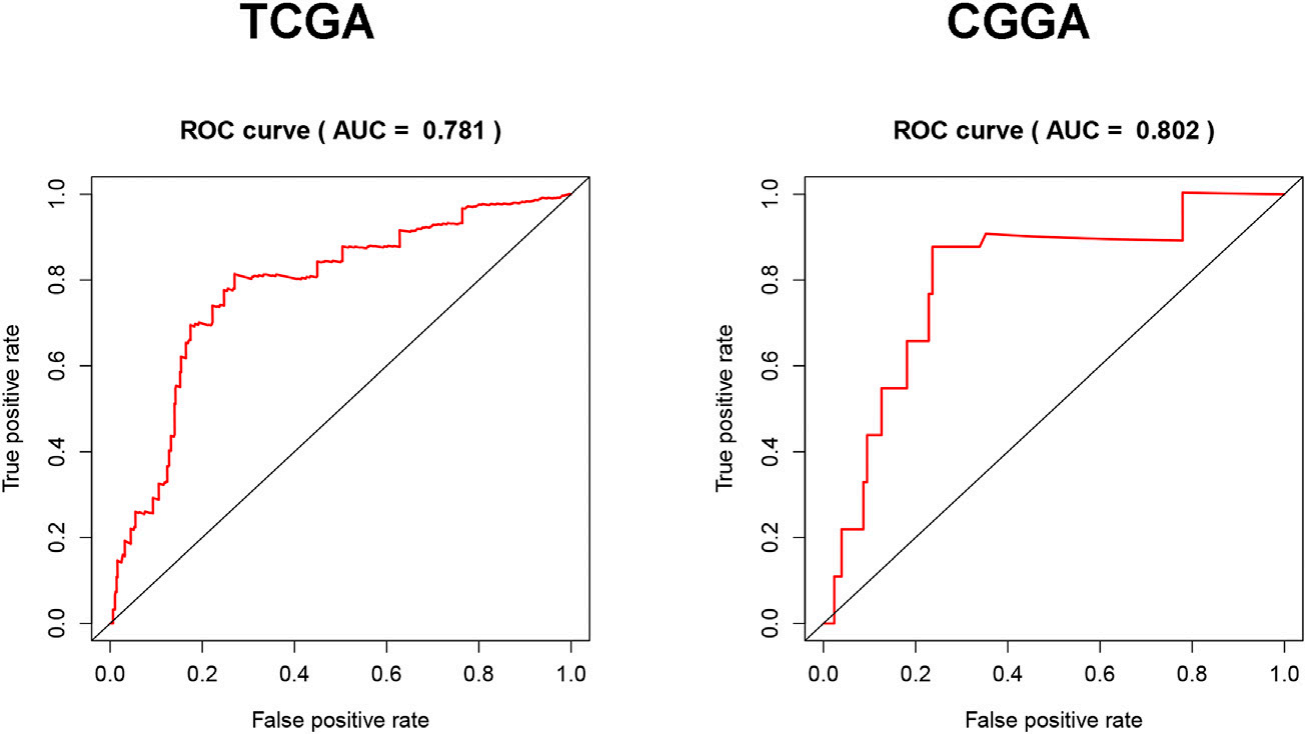
Diagnosis value of FAM20C expression in LGG analysis. **(A)** ROC curve for FAM20C expression in LGG tissues in TCGA database; **(B)** validation of FAM20C diagnosis value in CGGA database. ROC, receiver operating characteristic.

To evaluate the diagnostic value of Fam20C for LGG, TCGA RNA-seq data were employed to draw the ROC curve. The area under the ROC curve was 0.690, which had high diagnostic value (Figure 4A). This result was further verified with the CGGA data set, and the area under the ROC curve was 0.778 (Figure 4B).

### Functional Enrichment Analysis

**FIGURE 5:**
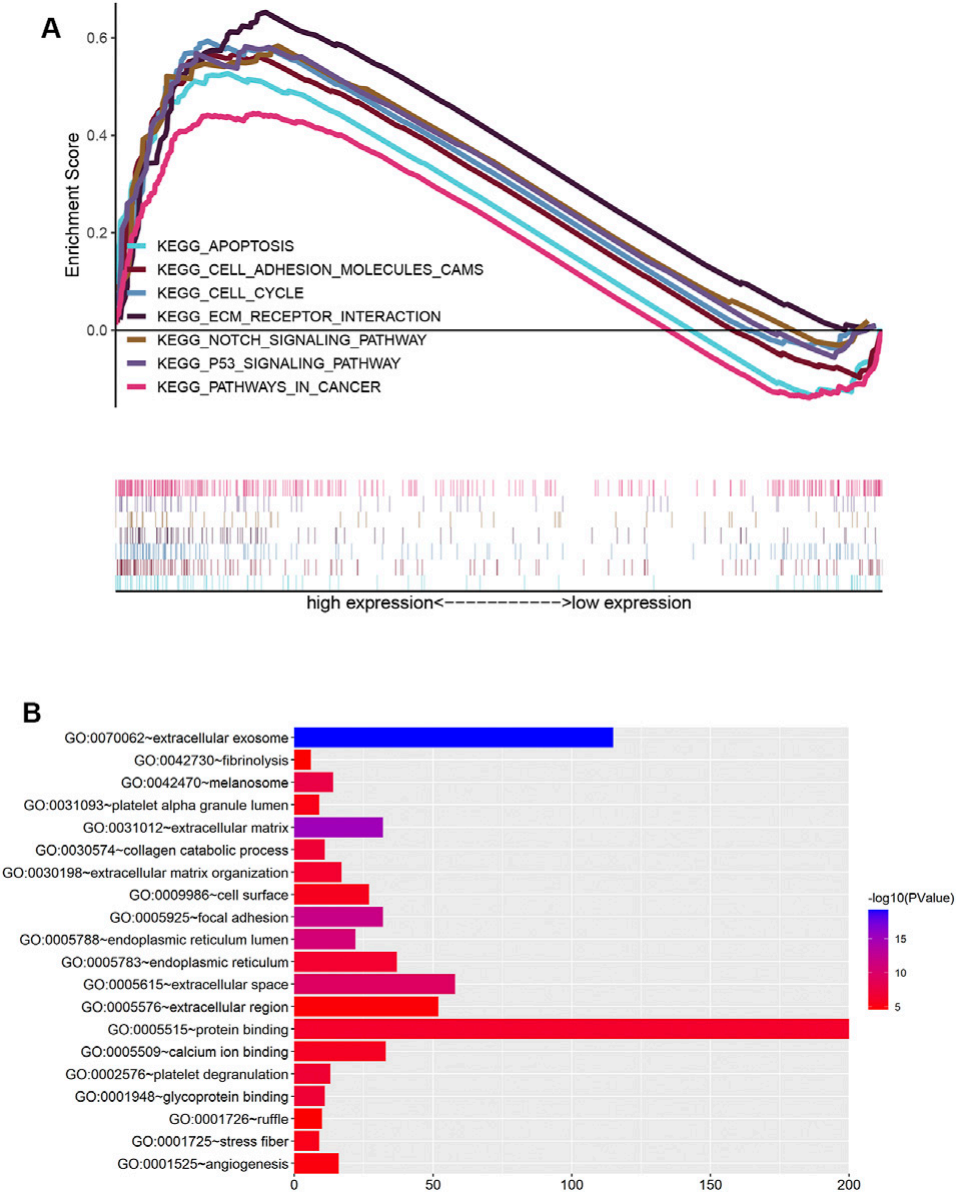
Functional enrichment analysis of Fam20c in LGG. **(A)** Gene Ontology enrichment analysis; **(B)** enrichment plots from GSEA. KEGG, Kyoto Encyclopedia of Genes and Genomes.

To clarify the functions and signaling pathways of genes co-expressed with Fam20C, we performed GO and KEGG enrichment analyses. GO analysis results showed that co-expressed genes were mainly closely related to the biological process of extracellular matrix remodeling (Figure 5A). KEGG analysis showed that co-expressed genes were mainly enriched in extracellular matrix receptor interactions, cell adhesion, apoptosis, cancer pathways, P53 signaling pathways, NOTCH signaling pathways, and cell cycle signaling pathways (Figure 5B).

### Fam20C Was Also Overexpressed in Our Cohort

**FIGURE 6:**
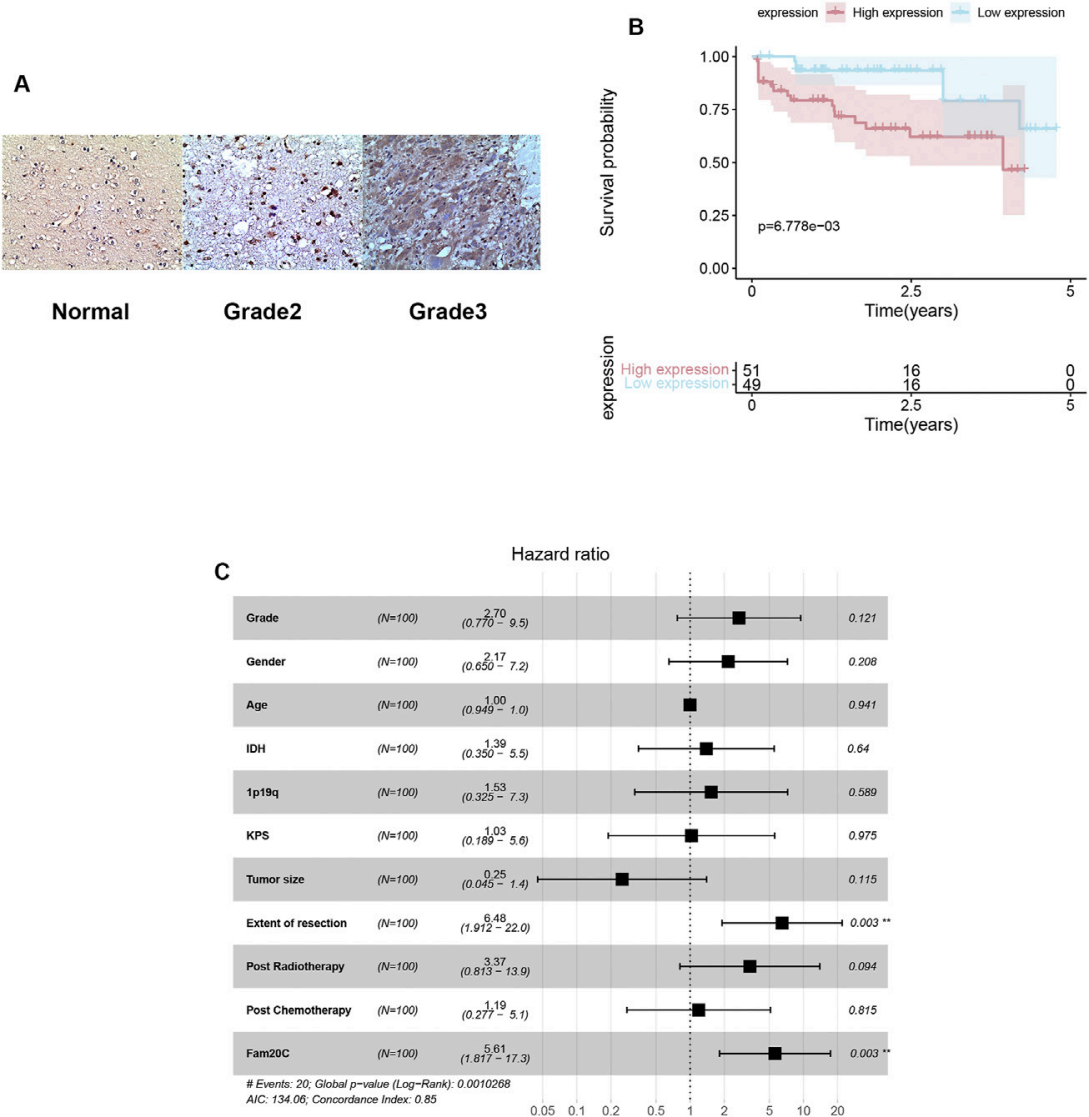
Expressions, immunohistochemistry and multivariate Cox analysis of Fam20c in our cohort. **(A)** Representative figures of FAM20C immune-staining in our clinical LGG samples (200X; grade II: *n* = 60, grade III: *n* = 40, normal: *n* = 3); **(B)** Kaplan–Meier curve evaluating the correlation between FAM20C protein expression and LGG patients’ survival (FAM20C low vs high, low *n* = 51, high *n* = 49, *p* < 0.001; Log rank test). **(C)** Multivariate Cox analysis evaluating independently predictive ability of Fam20c for OS.

To further verify the expression of Fam20C in our cohort, we detected its expression in our clinical samples and found that Fam20C was significantly overexpressed in grade 3 tumors (Figure 6A). Higher Fam20C expression levels were also correlated with a worse OS in our cohort (Figure 6B).

Univariate and multivariate Cox analyses were utilized to evaluate the independent prognostic values of Fam20C expression in LGG patients. The univariate analysis results showed that high Fam20C expression was significantly correlated with a shorter OS (HR = 6.39, 95% CI: 1.86–21.86, *p* = 0.003). Other variables related to poor survival included IDH 1p19q and extent of resection (Supplementary Table S4). Multivariate analysis showed that high expression of Fam20C in LGG patients was independently associated with a significant decrease in OS (Figure 6C and Supplementary Table S4).

## Discussion

Glioma is one of the most common primary malignant tumors in the nervous system. It arises from active glial cells in the brain, including astrocytes, oligodendrocytes, and ependymal cells. Although the prognosis of lower-grade glioma is better than that of glioblastoma, there are still some lower-grade gliomas with a poor prognosis and a short survival time, and 70% of low-grade patients undergo a high-grade transformation within 10 years. Therefore, early diagnosis and accurate prognostic biomarkers are essential for improving the prognosis of patients with LGG.

In recent years, a class of secreted kinases have been newly discovered that are involved in the regulation of many important physiological reactions. The Fam20 kinase family includes Fam20A, Fam20B, and Fam20C (Nalbant et al., 2005; Zhang et al., 2018). Fam20C is a casein kinase enriched in the Golgi apparatus that modulates many downstream substrates through protein phosphorylation and plays an important role in the formation of the secretome of tumor cells. However, its diagnostic and prognostic value in cancer is still unclear. Our results provide insights for further understanding the pathological role of Fam20C in promoting tumor growth and invasion and its potential value as a diagnostic and prognostic marker for LGG.

Fam20C protein kinase has a significant promotion effect on the metastasis and invasion of triple-negative breast cancer (Tagliabracci et al., 2015). Fam20 is also a potential target gene related to the pathogenesis of early lung adenocarcinoma (Kang 2013). Therefore, we speculate that the expression of Fam20C may affect the survival of patients through promoting the progression of tumor cells. However, the expression of Fam20C in cancer and its effects on other important aspects, such as tumor cell metastasis, still lack consensus. It has been previously reported that insulin-like growth factor binding protein 7 (IGFBP-7) regulates the migration of glioma cells through the AKT-ERK pathway, thereby playing an important role in the growth and migration of gliomas (Jiang et al., 2008). Adult diffuse glial tumor GWAS contains variants of D2HGDH and Fam20C in different molecular subtypes. In IDH mutant gliomas, the nine variants located on chromosome two of D2HGDH and those in its vicinity are all significant genome-wide (Eckel-Passow et al., 2020).

In this study, we systematically detected the expression level of Fam20C in different types of cancer in the TCGA database. Based on the available evidence, our results indicated that Fam20C expression was elevated in breast cancer. In addition, Fam20C was also overexpressed in five other cancers, such as glioma, meningioma, and kidney cancer, and Fam20C overexpression was associated with higher-grade gliomas.

At present, the biological functions of Fam20C and its mechanism of action in tumorigenesis have rarely been reported. The phosphorylated substrate of Fam20C is related to tumor cell apoptosis and migration and can accelerate the process of tumor metastasis by activating matrix metalloproteinases (MMPs). In this study, the Fam20C-related signaling pathways activated in LGG were mainly enriched in extracellular matrix receptor interactions, cell adhesion, apoptosis, cancer pathways, P53 signaling pathways, NOTCH signaling pathways, and the cell cycle, which further stimulated tumor proliferation and invasion.

Biomarkers are biological characteristics that could be objectively measured or evaluated. They may be employed as indicators of biological and pathological processes, or reflect the results of treatment methods, which are mainly used for disease prevention, diagnosis, treatment, prognosis, and drug development. Fam20C was an effective target for the treatment of triple-negative breast cancer. Fam20C inhibitor induced apoptosis of TNBC cell line (MDA-MB-468) and potentially inhibited cell migration (Qin et al., 2016). In our study, Fam20C expression was detected in postoperative pathological specimens of resectable glioma patients. Our present data has demonstrated that Fam20c may be a protentional prognostic marker for LGG. There is no research on whether Fam20C was highly expressed in the serum of LGG patients, we will reconsider and complete this topic in the future.

A Fam20C inhibitor induced cell apoptosis through the mitochondrial pathway and had the potential to inhibit cell migration (Qin et al., 2016; Zhao R. et al., 2021). Shaonan Du et al. found that Fam20C may serve as a predictive protein and a therapeutic target for GBM (Du et al., 2020). However, there have been few studies on the Fam20C gene in LGG. Hence, we further investigated whether Fam20C could be used as a diagnostic and prognostic marker for LGG. The ROC curve showed that the expression of Fam20C had a high diagnostic value for LGG. In addition, the Kaplan-Meier curves showed that high expression of Fam20C mRNA in LGG patients was significantly associated with a poor OS. In addition, univariate and multivariate Cox analyses showed that high Fam20C expression was an independent risk factor for a poor OS of LGG patients. In summary, our research showed that Fam20C was over-expressed in LGG and was correlated with more aggressive tumors and a worse prognosis. Our results showed that Fam20C is a promising biomarker for LGG diagnosis and prognosis.

## Conclusion

In conclusion, we established a potential prognostic and diagnostic signature for LGG patients based on two databases (TCGA and CGGA) and clinical samples. This biomarker could efficiently stratify the LGG patients into two groups with distinct survival differences. Moreover, we identified the potential signaling pathways of Fam20C in LGG patients. Overexpression of Fam20C was correlated with progressive malignancy and poor survival of LGG patients and was associated with significant enrichment of extracellular matrix receptor interactions, cell adhesion and apoptosis in LGG. Taken together, our results suggest that Fam20C inhibition could be a potential therapeutic target to prevent LGG progression.

## Data Availability

The datasets presented in this study can be found in online repositories at: http://github.com/fengjing0314/jing. The names of the repository/repositories and accession number(s) can be found in the article/Supplementary Material.
