# Supplementary figures and images for: Fam20C Overexpression Predicts Poor Outcomes and is a Diagnostic Biomarker in Lower-Grade Glioma
Source: Front Genet. 2021 Dec 14;12:757014. doi: 10.3389/fgene.2021.757014 (PMC8712682; doi:10.3389/fgene.2021.757014)

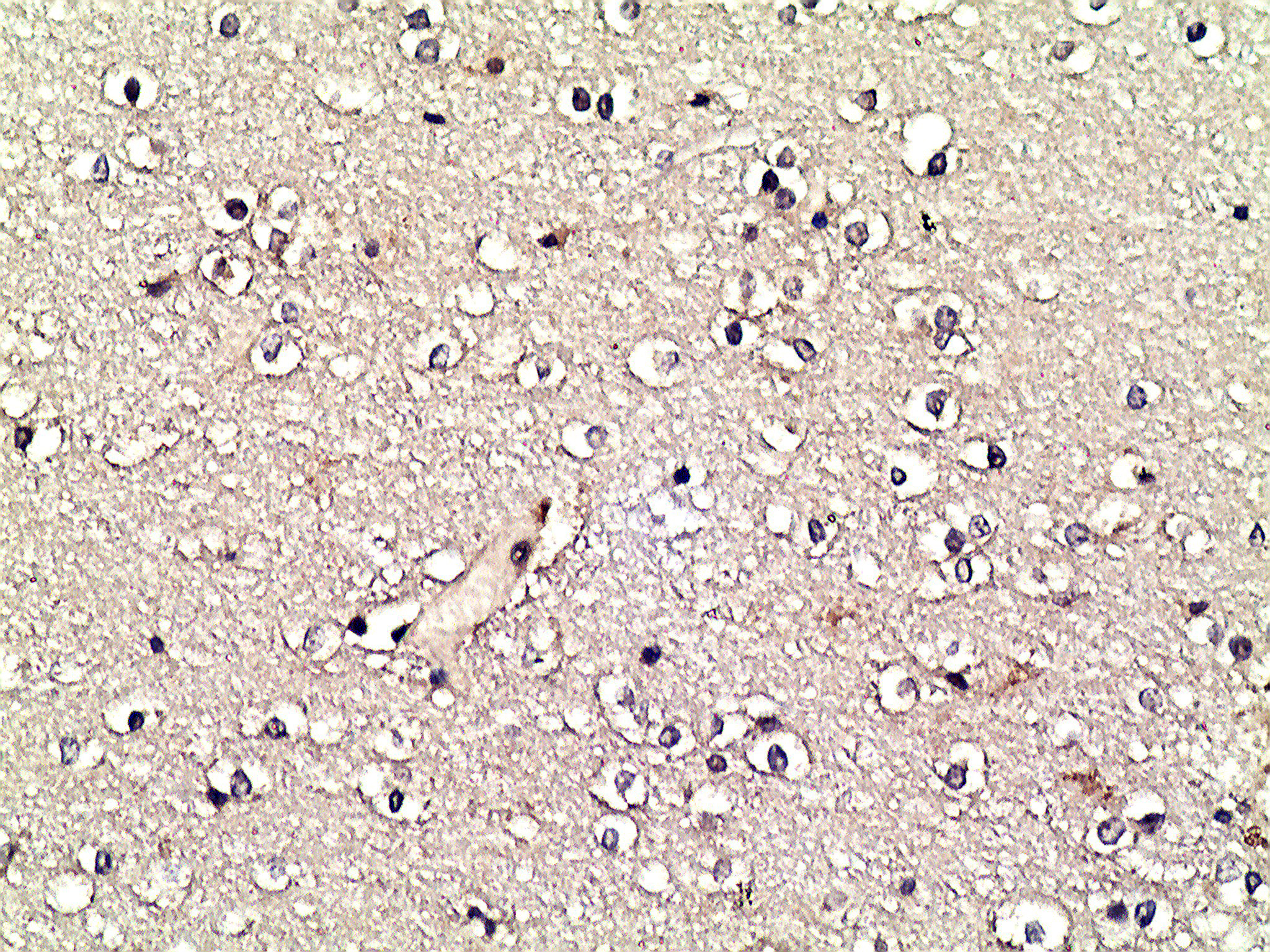

Supplement: Supplementary file 1 [file Image3.JPEG]

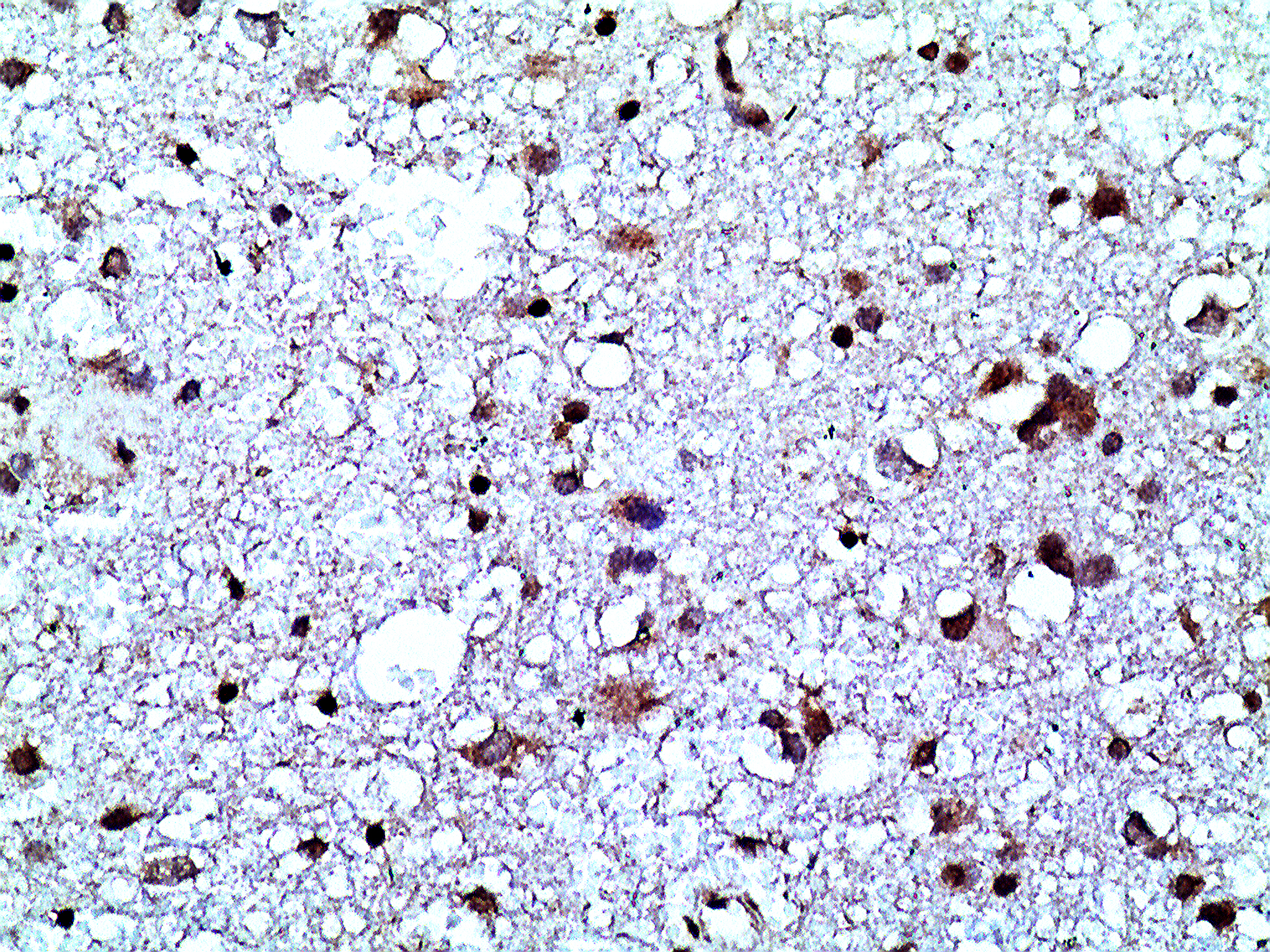

Supplement: Supplementary file 2 [file Image1.JPEG]

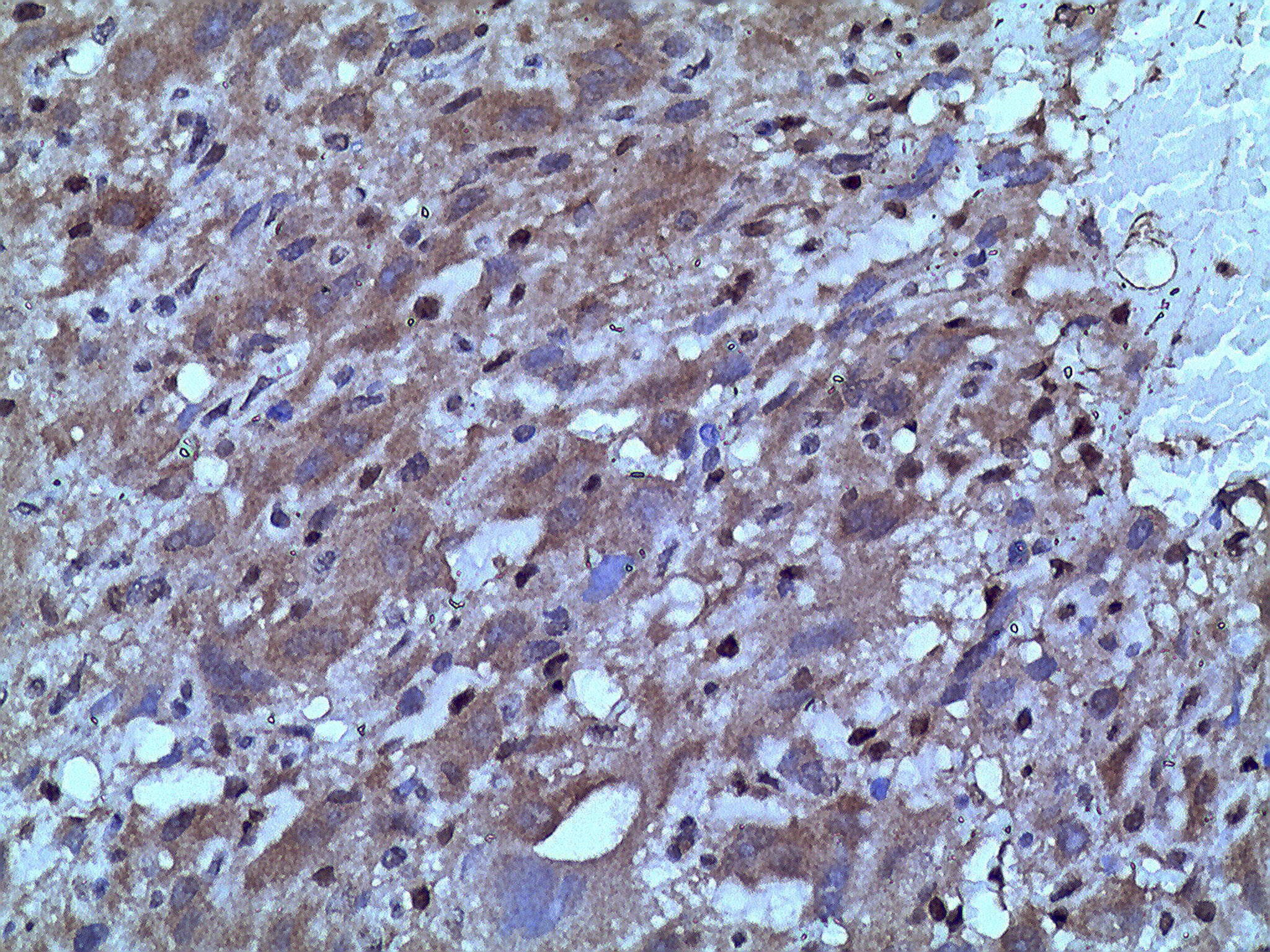

Supplement: Supplementary file 3 [file Image2.JPEG]
