# Supplementary material for: Fam20C Overexpression Predicts Poor Outcomes and is a Diagnostic Biomarker in Lower-Grade Glioma
Source: Front Genet. 2021 Dec 14;12:757014. doi: 10.3389/fgene.2021.757014 (PMC8712682; doi:10.3389/fgene.2021.757014)
Supplement: Supplementary file 4 [file DataSheet1.docx]

Supplementary Material

**Supplementary Table 1.** Clinical Characteristics of Patients in TCGA, CGGA Database and Our Cohort(Cont)

| **Variate** | **TCGA（N%）** | **CCGA（N%）** | **Our cohort（N%）** |
| --- | --- | --- | --- |
| **Age** |  |  |  |
| ＜40 y | 240（46.69%） | 67（50.76%） | 40（40%） |
| ≥40 y | 274（53.31%） | 65（49.24%） | 60（60%） |
| **Gender** |  |  |  |
| Male | 284（55.25%） | 81（61.36%） | 71（71%） |
| Female | 230（44.75%） | 51（38.64%） | 29（29%） |
| **Grade** |  |  |  |
| 2 | 249（48.44%） | 87（65.91%） | 60（60%） |
| 3 | 265（51.56%） | 45（34.09%） | 40（40%） |
| **IDH** |  |  |  |
| mutation | - | 98（74.24%） | 78（78%） |
| wildtype | - | 34（25.76%） | 22（22%） |
| **1p19q** |  |  |  |
| codeletion | - | 108（81.82%） | 50（50%） |
| no-codeletion | - | 24（18.18%） | 50（50%） |

| **Variate** | **TCGA（N%）** | **CCGA（N%）** | **Our cohort（N%）** |
| --- | --- | --- | --- |
| **KPS** |  |  |  |
| ≥60 | - | - | 87（87%） |
| ＜60 | - | - | 13（13%） |
| **Tumor size** |  |  |  |
| ≥6cm | - | - | 20（20%） |
| ＜6cm | - | - | 80（80%） |
| **Extent of resection** |  |  |  |
| complete resection | - | - | 64（64%） |
| residual | - | - | 36（36%） |
| **Post radiotherapy** |  |  |  |
| Yes | - | - | 62（62%） |
| No | - | - | 38（38%） |
| **Post chemotherapy** |  |  |  |
| Yes | - | - | 58（58%） |
| No | - | - | 42（42%） |

**Refer to Supplementary Table 1(continued)**

**Supplementary Table 2.** Univariate and Multivariate COX Regression Analysis of FAM20C for LGG Patients Overall Survival in The Cancer Genome Atlas Database

| overall survival | | | | | |
| --- | --- | --- | --- | --- | --- |
|  | univariate |  | multivariate | | |
| variable | HR(95 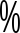CI) | p |  | HR | p |
| Age |  |  |  |  |  |
| ≥40 (n=269) vs. <40 (n=236) | 1.06(1.04-1.08) | **<0.001** |  | 1.06(1.04-1.08) | **<0.001** |
| Gender |  |  |  |  |  |
| Male (n=275) vs. female (n=226） | 1.06(0.73-1.55) | 0.762 |  | 1.05(0.72-1.55) | 0.789 |
| Grade |  |  |  |  |  |
| Grade 2(n=245) vs. Grade 3 (n=260) | 3.12(2.06-4.72) | **<0.001** |  | 2.00(1.29-3.10) | **0.002** |
| FAM20C | 1.02(1.01-1.03) | **<0.001** |  | 1.73(1.38-2.18) | **<0.001** |

**Supplementary Table 3.** Univariate and Multivariate COX Regression Analysis of FAM20C for LGG Patients Overall Survival in The Chinese Glioma Genome Atlas Database

|  | overall survival | | | | |
| --- | --- | --- | --- | --- | --- |
|  | univariate | |  | multivariate | |
| **variable** | HR (95%CI) | *p* |  | HR (95%CI) | *p* |
| **Grade**  **Age** | 0.73(0.60-0.89)  1.58(0.96-2.59) | **0.002**  0.069 |  | 3.12(1.82-5.37)  0.96(0.56-1.64) | **<0.001**  0.869 |
| **Gender** | 0.67(0.41-1.09) | 0.107 |  | 1.69(1.01-2.82) | 0.046 |
| **IDH** | 3.37(2.02-5.61) | **<0.001** |  | 1.13(0.60-2.12) | 0.705 |
| **1p19q** | 6.57(3.2-13.40) | **<0.001** |  | 5.56(2.61-11.87) | <**0.001** |
| **Fam20C** | 1.01(1.00-1.01) | **0.001** |  | 1.30(1.03-1.65) | **0.028** |

**Supplementary Table 4.** Univariate and Multivariate COX Regression Analysis of FAM20C for LGG Patients Overall Survival in Our Cohort Database

|  | overall survival | | | | |
| --- | --- | --- | --- | --- | --- |
|  | univariate | |  | multivariate | |
| **variable** | HR (95%CI) | *p* |  | HR (95%CI) | *p* |
| **Grade**  **Age** | 1.74(0.71-4.31)  1.02(0.99-1.06) | 0.226  0.230 |  | 2.69(0.77-9.45)  0.99(0.65-7.23) | 0.121  0.941 |
| **Gender** | 1.26(0.47-3.38) | 0.642 |  | 2.17(0.65-7.23) | 0.208 |
| **IDH** | 2.85(1.09-7.45) | **0.033** |  | 1.39(0.35-5.52) | 0.640 |
| **1p19q** | 3.76(1.21-11.76) | **0.023** |  | 1.53(0.32-7.25) | 0.589 |
| **KPS** | 0.90(0.25-3.32) | 0.877 |  | 1.03(0.19-5.57) | 0.975 |
| **Tumor size** | 0.57(0.16-1.98) | 0.374 |  | 0.25(0.04-1.40) | 0.115 |
| **Extent of resection** | 2.52(1.00-6.34) | **0.049** |  | 6.48(1.91-21.96) | **0.003** |
| **Post radiotherapy** | 2.31(0.91-5.91) | 0.077 |  | 3.37(0.81-13.95) | 0.094 |
| **Post chemotherapy** | 1.19(0.27-5.12) | 0.815 |  | 1.19(0.27-5.12) | 0.815 |
| **Fam20C** | 6.39(1.86-21.86) | **0.003** |  | 5.60(1.82-17.32) | **0.003** |


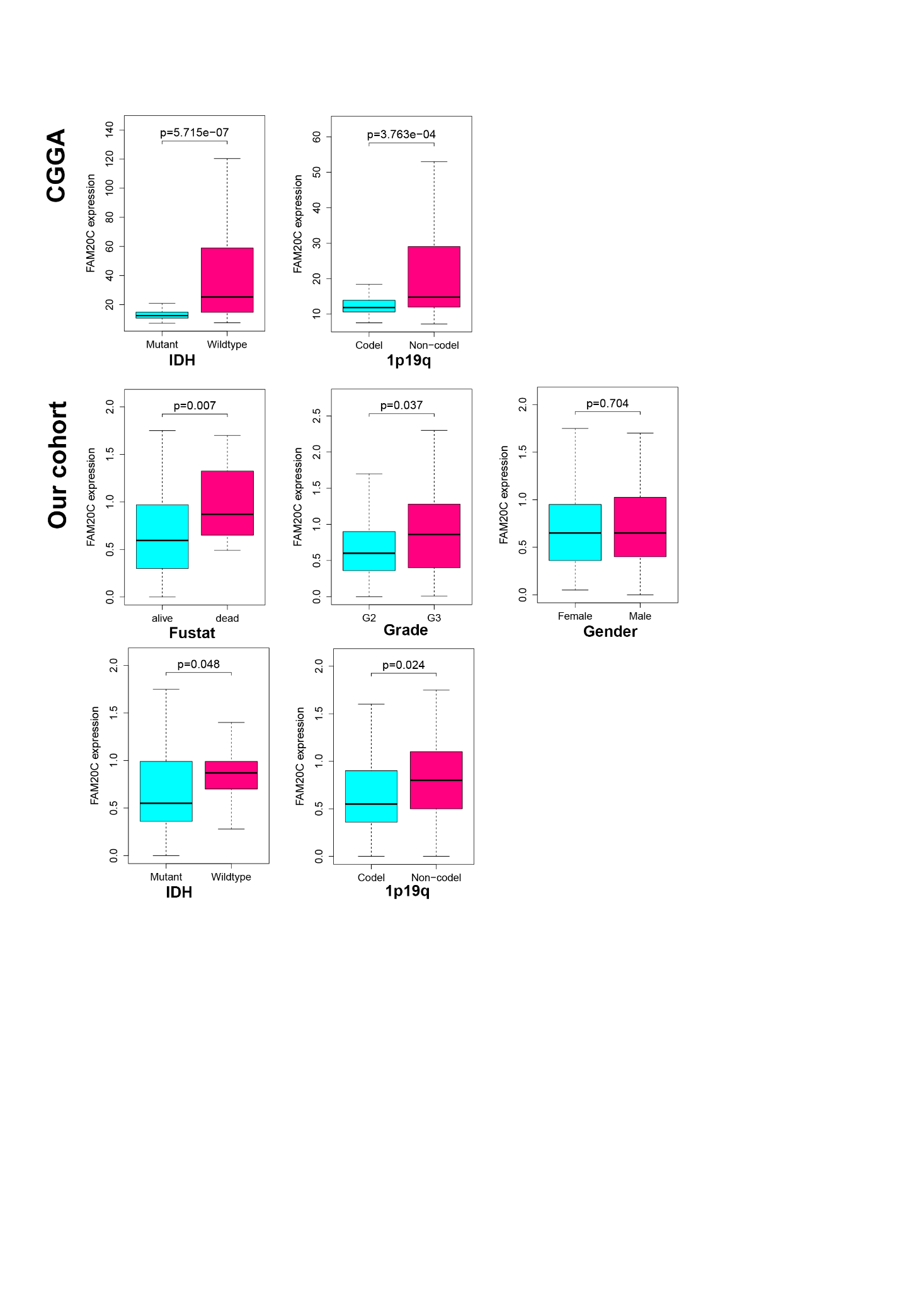


**Supplementary Figure 1.** Relationship between IDH mutation/1p19q deletion status and FAM20C expression in the CGGA database and our cohort samples.
